# A Comparative Evaluation of the Caloric Intake and Economic Efficiency of Two Types of Homogenized Diets in a Hospital Setting

**DOI:** 10.3390/nu15224731

**Published:** 2023-11-09

**Authors:** Camilla Crippa, Sofia Matteucci, Manuela Pastore, Emanuela Morenghi, Erica Starace, Giulia De Pasquale, Gabriella Pieri, Fanny Soekeland, Stefano Maria Gibbi, Giuliana Lo Cricchio, Andrea Zorloni, Beatrice Mazzoleni, Stefano Mancin

**Affiliations:** 1 IRCCS Humanitas Research Hospital, 20089 Rozzano, Italy; camilla.crippa@humanitas.it (C.C.); sofia.matteucci@humanitas.it (S.M.); manuela.pastore@humanitas.it (M.P.); emanuela.morenghi@humanitas.it (E.M.); erica.starace@humanitas.it (E.S.); giulia.depasquale@humanitas.it (G.D.P.); gabriella.pieri@humanitas.it (G.P.); giuliana.locricchio@asst-santipaolocarlo.it (G.L.C.); 2 Department of Biomedical Sciences, Humanitas University, 20072 Pieve Emanuele, Italy; andrea.zorloni@st.hunimed.eu (A.Z.); beatrice.mazzoleni@hunimed.eu (B.M.); 3 School of Health Professions, University of Applied Sciences, 3008 Bern, Switzerland; fanny.soeke@gmail.com; 4 School of Pharmacy, Department of Drug Science University of Pavia, 27100 Pavia, Italy; stefanomaria.gibbi01@universitadipavia.it; 5 Operating Room Unit Humanitas San Pio X, 20159 Milan, Italy

**Keywords:** hospital nutrition, diet, malnutrition, feeding difficulties

## Abstract

The prevalence of malnutrition is increasing globally due to factors such as age-related pathological conditions and diseases that impact food and beverage intake. In hospital settings, older adult patients often require homogenised diets, which can lead to malnutrition due to poor palatability and limited variety. This study compared the Standard Homogenised Diet (HSD) and a Modified Homogenized Diet (HMD) proposed in a tertiary hospital in Northern Italy. A retrospective and observational design was used to analyse data from 86 adult patients with various conditions requiring a homogenised diet. The primary goal was to compare food intake, rheological characteristics, and palatability of the two diets. The secondary objective was to evaluate the economic impact by comparing costs and quantifying food waste from unused meals. Patients on HMD had a median daily caloric intake of 852 kcal (IQR 787–926 kcal) compared to 631 kcal (IQR 506–797 kcal) in the HSD group. Taste, texture, palatability, and ease of intake for HMD outperformed HSD with scores such as 3.7 ± 0.6 vs. 2.5 ± 0.4 for taste. Economically, HMD was EUR 0.53 less expensive per day than HSD, and food wastage costs were significantly lower for HMD (EUR 2.66 ± 0.81) than HSD (EUR 4.66 ± 1.27). Overall, HMD presented substantial benefits in patient satisfaction and cost-efficiency. This insight may aid diverse care settings to enhance meal acceptance and nutritional intake for patients needing homogenised diets.

## 1. Introduction

Malnutrition prevalence is increasing worldwide due to aging of the population and the increasing prevalence of age-related pathological conditions [1], as well as diseases that can directly influence the intake of food and drink (for example, cancer-induced dysphagia, dementia, dysphagia, edentulism) [2]. These conditions significantly increase the risk of malnutrition, compromising the function and recovery of the body [3,4]. Malnutrition can be defined as a state resulting from a lack of intake or uptake of nutrients that lead to altered body composition and body cell mass, resulting in diminished physical and mental function and impaired clinical outcome from disease [5]. Among hospitalised older adult individuals, protein–calorie, mineral, and vitamin malnutrition are particularly common and, in addition to other predisposing factors to sarcopenia, lead to increased muscle mass with aging [6,7]. Institutionalised patients suffering from dysphagia form a high percentage, ranging from 25 to 85% [8]. Dysphagia is a common problem among older adults [9,10] and is generally defined as swallowing difficulty caused by neurological diseases [11]. On the other hand, presbyphagia refers to age-related changes that affect the swallowing mechanism in the older adult population, which are characterised by nervous, skeletal, and muscle systems [12,13]. Additionally, malnutrition depends on the presence of specific situations such as edentulia, defined as complete or partial loss of teeth [14]. These conditions make chewing and swallowing solid foods difficult, often leading older adult patients to prefer softer or liquid foods. This can lead to the choice of easier-to-eat foods or the need for dentures or modifications in the texture of the food [15].

Faced with these issues, older adult patients often require a homogenised diet, frequently dealing with the problem of palatability of food [16]. The homogenised consistency and limited variety of foods can negatively affect the visual appearance, taste, and overall texture of the meal, which can lead to decreased adherence and malnutrition [17]. In fact, many studies have shown a lower caloric intake resulting from modified diets [17,18]. In this sense, it is crucial to consider the importance of the role of the hospital diet in the prevention and treatment of hospital malnutrition in this population. The offer of more palatable and varied foods with a texture specifically designed for patients with dysphagia, presbyphagia, edentulism, and other chewing difficulties could increase the protein–calorie and, above all, fluid intake of the diet, thus reducing the risk of malnutrition and dehydration in hospitalised older adult individuals [16,19,20]. Considering this, it is essential to not only assess the nutritional status of patients who experience difficulties in oral food intake and requiring a homogenised diet, but also to measure adherence to the prescribed diet therapy and evaluate the palatability of the diet itself. Only by paying attention with care and attention to the nutritional needs of these patients can positive results be achieved in the prevention and treatment of hospital malnutrition.

## 2. Materials and Methods

### 2.1. Study Design

The study was conducted using a retrospective and observational design. Data was obtained from the electronic medical records of the IRCCS Humanitas Research Hospital, Rozzano, Milan. Access to medical records was requested and authorised by the Medical and Health Directorate and the Ethics Committee CET Lombardia 5, with authorisation number 22/23. The study methods were in accordance with the Strengthening the Reporting of Observational Studies in Epidemiology (STROBE) checklist [21] (Supplementary File).

### 2.2. Objectives of the Study

**Figure 1:**
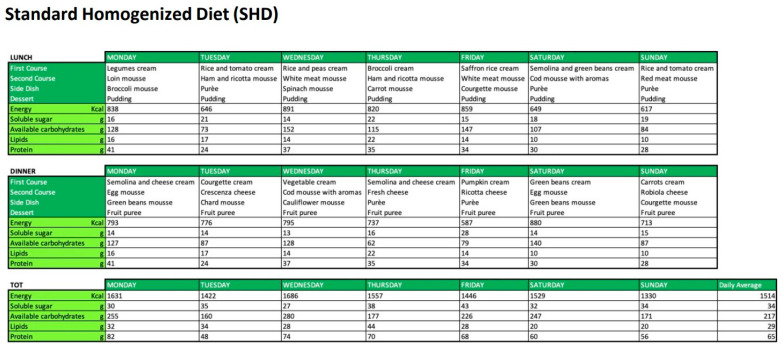
Menus and relative nutritional tables of Homogenised Standard Diet (HSD).

**Figure 2:**
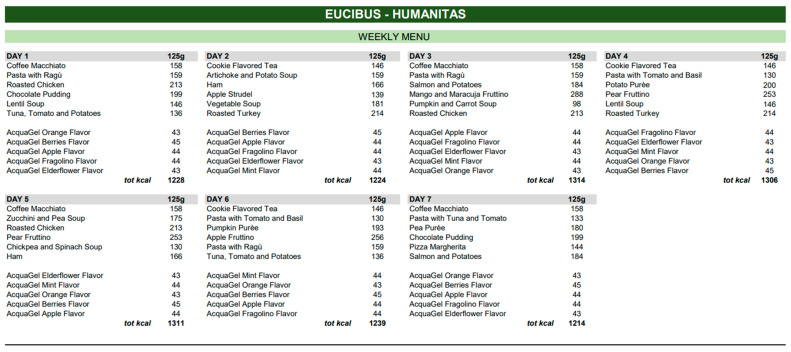
Menus and relative nutritional tables of Homogenised Modified Diet (HMD).

This study will evaluate two types of homogenised diets proposed in a tertiary hospital in Northern Italy, during the period between January and March 2023 (Figure 1 and Figure 2): (1) Homogenised Standard Diet (HSD) provided by the hospital catering service, consisting of pureed or packaged homogenized foods; (2) Homogenized Modified Diet consisting of foods formulated with natural ingredients, top quality and controlled supply chain, with a creamy consistency, density, and high lubricating action for safe swallowing (HMD). These foods were prepared in storage packages outside the cold chain, preserving the organoleptic and nutritional properties of the products. The prescribed diets, although differing in menu and presentation, were similar in terms of nutritional content and composition. Both diets were balanced in terms of macro- and micronutrient distribution, ensuring a similar caloric intake and adhering to the daily caloric requirements recommended. These caloric requirements are based on the reference levels provided by the Italian Ministry of Health, which offers detailed guidelines on the recommended amounts of carbohydrates, proteins, fats, and other essential nutrients. The key difference between HSD and HMD lies not only in their presentation and preservation, but also in the quality of ingredients used. While the HSD represents a traditional diet offered by hospital catering services, the HMD stands out due to its utilization of high-quality natural ingredients and its packaging, which allows for storage outside of the cold chain, ensuring prolonged freshness and palatability. Furthermore, the HMD, in comparison to the HSD, has a structure and composition that could facilitate easier consumption, especially in individuals with cognitive and functional deficits, such as the older adults. Similarly, to the HSD, the HMD diet was standardized, administered based on the various pathologies and comorbidities of the patients, and prescribed by the attending physician. Both diets were designed for a “clinical” setting and adhered to strict safety standards.

The aim was to ensure that, regardless of the chosen diet, each patient received optimal nutritional intake consistent with these recommendations [22]. The primary objective of the study was to evaluate the food intake of the two proposed homogenised diets, oral hydration, rheological characteristics, and palatability of the diets. Subsequently, the obtained data was correlated with clinical variables of interest, such as age, underlying disease, comorbidity, reason for prescribing the homogenised diet, and nutritional status. The secondary objective of the study was to assess the economic impact by comparing the two diets and evaluating the average daily costs, taking into account and quantifying the food waste from uncooked meals.

### 2.3. Inclusion and Exclusion Criteria

Inclusion criteria: Adult patients, hospitalized in the Internal Medicine and Neurology departments of the IRCCS Humanitas Research Hospital of Rozzano between January and March 2023, who had been prescribed a homogenized diet due to feeding difficulties due to swallowing disorders (including dysphagia), chewing difficulties, neurological problems, edentulism, or any other condition requiring the prescription of a homogenized diet. All patients had to have no clinical contraindications to oral food intake and had to be independent in their diet. In cases of partial independence or complete inability to self-feed, patients were supported by caregivers, family members, or caregivers.

Since several factors could influence food intake, including physical activity levels, mental status, and cognitive function, upon admission, patients’ physical activity level was assessed by nursing staff using specific forms. Those identified with impaired self-administration of meals were cared for by support staff. For mental status and cognitive function, we used the Glasgow Coma Scale (GCS), a clinical tool that assesses a patient’s level of consciousness, providing a score between 3 (deeply unconscious) and 15 (fully conscious); this allowed us to exclude patients with severe cognitive impairment.

The presence of dysphagia, identified during hospitalization, was diagnosed through the 3OZ water swallow test. This test evaluates a patient’s ability to swallow a certain volume of water without showing signs of respiratory difficulty or choking. Dysphagia grades, ranging from 1 to 4, respectively indicate mild to severe dysphagia. For our study, we included only patients with dysphagia levels 2 and 3, corresponding to those who need to feed on a homogenous consistency diet. Patients with grade 1 dysphagia, having no significant issues, and those with grade 4 dysphagia, as they present severely compromised swallowing and might need more complex dietary interventions or alternatives to oral nutrition, were excluded. In the selected patients, the diagnosis and degree of dysphagia were further confirmed by an in-depth evaluation by a speech therapist and the treating physician. The decision to only include patients with dysphagia of grade 2 and 3 was also driven by the need to assess the two dietary options in a uniform group of subjects.

Exclusion criteria: Inability to take oral nutrition; complications such as significantly alter the neurological status (patients with a Glasgow Coma Scale of 8 or below indicating moderate to severe impairment in consciousness); non-homogenised diet; patients who do not meet inclusion criteria.

In this study, 86 eligible patients were enrolled according to the previously stated inclusion and exclusion criteria. An initial sample size calculation could not be performed due to the explorative nature of this retrospective observational study. The number of patients was determined based on the number of prescribed diets in the considered period for respective study.

### 2.4. Data Collection

The data was collected through a dedicated anonymised electronic database protected by a password. Specifically, data was obtained retrospectively through the analysis of electronic medical records and according to the declared inclusion/exclusion criteria. The prescription of the specific homogenized diet, be it HMD or HSD, was determined based on the medical dietary prescription, and not through randomized allocation. The collected data pertained to the phases of acceptance upon medical and nursing admission and adhered to the clinical standards of the hospital facility. Even though this study is of a retrospective nature, our research implemented specific methodological measures to minimize potential biases. Healthcare professionals, following the standard procedure used in our hospital, recorded the actual food intake using dedicated forms available in the electronic medical records. The assessment of palatability and rheological characteristics of the foods was evaluated for the specified period during which the HMD diet was introduced. This data collection process, which began from patient admission, covered different phases:

(1) Hospital admission: age, sex, body mass index (BMI), plasma albumin level, underlying disease, comorbidities (Type I-II diabetes mellitus, cardiopathy, chronic kidney disease stage I–IV, chronic obstructive pulmonary disease, cancer).
(2) Within 72 h of hospitalization or of the first prescription of the homogenized diet: evaluation of fluid and food intake. This survey was carried out by the health workers of the wards and recorded in the electronic medical record; The daily millilitres of liquids consumed, the daily kilocalories, and the percentage of food consumed were considered. This assessment is carried out as a standard hospital procedure in order to identify possible alterations in patients’ water or food intake. For the purposes of this study, three days of evaluation were considered in order to make the collected data homogeneous in consideration of the different periods of hospitalization, which differed in the sample considered.
(3) After measuring water and food intake, two external evaluators (academic staff) carried out an evaluation of the rheological characteristics of the foods consumed and the palatability of the homogenized diet taken using an evaluation scale that considered variables such as taste, texture, palatability, practicality, and ease of use. Each variable was assigned a score according to a 5-point Likert scale (1 = insufficient, 2 = sufficient, 3 = good, 4 = excellent, 5 = exceptional). Both evaluators were unaware of the diet administered by the hospital catering service and had previously followed specific training conducted by a dietitian and a nutritionist in order to interview patients in a standardized way. Additionally, each assessment form was supported by a dedicated legend to interpret patient feedback and assign an assessment score. Data collection for this study was performed retrospectively and managed by an independent data manager, receiving data that was anonymized and devoid of any identifying information about either the patient or diet. This approach further ensured the integrity and accuracy of the collected data. The data obtained was then used to address the primary and secondary objectives. As this study is purely observational and retrospective, follow-up periods were not included.

### 2.5. Study Procedures

After the retrospective data collection, the gathered data was analysed, dividing the study into two distinct phases: a clinical phase and an economic phase. In the clinical phase of the study, both food and water intake data were aggregated. We assessed the average total caloric intake (in Kcal/day) and the percentage of intake, comparing the two administered diets (HSD vs. HMD). It is noteworthy that while the water in the form of gel in the HMD diet contained calories (as depicted in Figure 1), the HSD diet, in contrast, had no calories from the gel. This aspect was factored into the overall caloric intake assessment.

Furthermore, a second clinical evaluation identified the characteristics of the proposed homogenised diets to determine their rheological properties and palatability. Variables such as taste, texture, palatability, practicality, and ease of use were scored on a 5-point Likert scale (1 = insufficient, 2 = sufficient, 3 = good, 4 = excellent, 5 = exceptional). Subsequently, the obtained data were correlated with each other and a subsequent analysis was performed to evaluate possible correlations with clinical variables recorded at hospital admission, such as age, body mass index (BMI), plasma albumin level, underlying disease, and comorbidities (Type I–II diabetes mellitus, cardiopathy, chronic kidney disease, chronic obstructive pulmonary disease), as they could be considered confounding factors in the study data.

Moving on to the economic phase of the study, a detailed analysis was conducted with the aim of determining the cost differences between HSD and HMD, expressed in euros per total daily meal. This included understanding the extent of food waste, measured as the percentage of uneaten homogenized portions for each type of diet. To ensure accuracy and transparency in this assessment, the costs for the HSD and HMD were obtained upon request from the Hospital’s Directorate of Health and Department of Pharmacy who provided a detailed estimate. It is also interesting to note that there were no conflicts of interest or external financial incentives that could affect the objectivity of our study, as both diets were provided by the Food Service Department (HSD) and the Hospital Department of Pharmacy (HMD).

### 2.6. Statistical Analysis

Data were described as number and percentage, or mean and standard deviation, with a 95% confidence interval (95%CI), if necessary, or median and range, as appropriate. Differences between two types of nutrition were explored with chi square test or t student test or Mann–Whitney test, as appropriate. Association with food intake were evaluated with linear regression, in a univariate way, and with multivariate analysis, if necessary, considering ten patients per variable criterion. All analyses were performed with Stata version 17. The significance level was set to 0.05.

### 2.7. Acronyms

The acronyms used in this study encompass terms from various fields, including dietetics, healthcare institutions, epidemiology, medical conditions, and statistics. HSD and HMD represent the homogenised standard diet and the Homogenized Modified Diet, provided in the hospital setting. IRCCS stands for the Institute for Scientific Hospitalisation and Care. STROBE refers to the guidelines to strengthen the Reporting of Observational Studies in epidemiology. BMI, MUST, COPD, and CKD represent the Body Mass Index, Universal Malnutrition Screening Tool, Chronic Obstructive Pulmonary Disease, and Chronic Kidney Disease, respectively. IQR and Kcal denote the interquartile range and Kilocalories, respectively. All these acronyms were used to simplify and improve the readability of the text, each being defined at its first mention in the document.

## 3. Results

### 3.1. Demographic and Clinical Characteristics of the Sample

In our study, we retrospectively examined a sample of 86 participants, 55 patients in the HSD group and 31 in the HMD group. The demographic and clinical characteristics of these two groups are summarized in Table 1. The two groups did not show significant differences in terms of age, gender distribution, BMI, and albumin level. Furthermore, we observed no significant differences in disease profiles between the two groups, with around 70% of patients in each group having comorbidities, as well as the number of comorbidities per participant was similar between the two groups. Regarding specific health conditions, the prevalence of grade 2–3 dysphagia was 47.27% in the HSD group compared with 37.04% in the HMD group. However, the difference was not statistically significant (*p* = 0.479). Similarly, the Malnutrition Universal Screening Tool (MUST) score, an indicator of nutritional status, was nearly identical in the two groups.

### 3.2. Dietary Intake of the Homogenized Diets

A first analysis was carried out to measure the daily caloric intake between the two samples under study (HSD and HMD group). The group of patients taking the HSD diet showed a median daily caloric intake of 631 kcal (IQR 506–797 kcal). Furthermore, the range of daily caloric intake within this group extended from a low extreme of 222 kcal to an upper extreme of 1028 kcal.

In contrast, the HMD adherent group demonstrated a higher calorie intake profile. The median daily caloric intake in this group was quantified as significantly higher, 852 kcal (IQR 787–926 kcal). The distribution of daily caloric intake values within the HMD group ranged from 689 to 1028 kcal (*p* < 0.001).

**Figure 3:**
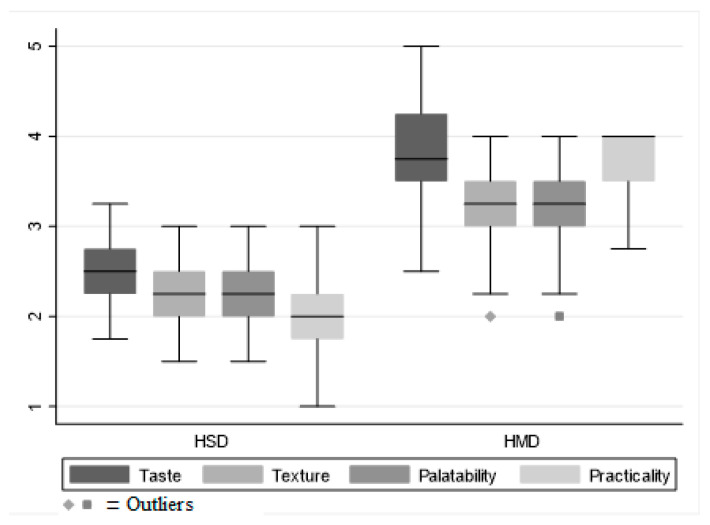
Box plot of rheological properties and palatability of homogenised diets.

A second evaluation considered rheological variables such as: taste, texture, palatability, and ease of food intake, also indicating differences in dietary experiences between the two groups, with statistically significant differences observed (*p* < 0.001) (Figure 3).

In all four characteristics evaluated: taste, texture, palatability, and practicality (Table 2), HMD scored higher on average than HSD. Specifically, the mean taste score for HMD was 3.7 ± 0.6, ranging from “good” to “very good” on the Likert scale. In contrast, HSD received a score of 2.5 ± 0.4, slightly higher than “sufficient” (*p* < 0.001), indicating a preference for the taste of HMD over HSD. Similarly, the structure was also evaluated more favourably in the HMD, with a mean score of 3.2 ± 0.5, compared to 2.3 ± 0.4 for the HSD (*p* < 0.001), as well as palatability, (3.2 ± 0.5 vs. 2.3 ± 0.4, *p* < 0.001). Furthermore, convenience, which included factors related to ease of consumption, received a higher score in the HMD group (3.7 ± 0.4 vs. 2.0 ± 0.4, *p* < 0.001) (Figure 3).

### 3.3. Food Intake and Clinical Variables

To address potential sources of bias, the study examined the correlation between daily calorie intake and several clinical variables of interest, including sex, age, BMI, plasma albumin, and comorbidities. Among the variables evaluated, sex was the only variable that had a significant correlation with daily calories intake. Male participants had a lower caloric intake (663 ± 213 kcal) than female participants (758 ± 187 kcal), with a regression coefficient of −96.8 (95%CI −182.6 to −10.9, *p* = 0.028). Other variables, including age, BMI, plasma albumin, presence and number of comorbidities, and presence of dysphagia did not show a significant correlation with daily caloric intake. Therefore, based on these results, these variables were not identified as potential sources of bias in the comparison of daily caloric intake between the HSD and HMD groups.

### 3.4. Economic Impact of Diets

In this study, we next evaluated the costs between HSD and HMD. The daily cost of the HSD was EUR 10.00, and the cost of the HMD was EUR 9.47. The costs quoted covered all meals and beverages per day with no restrictions including breakfast, lunch, dinner, and two snacks, plus liquids. For dysphagic patients, thickened water was provided as part of their diet.

**Figure 4:**
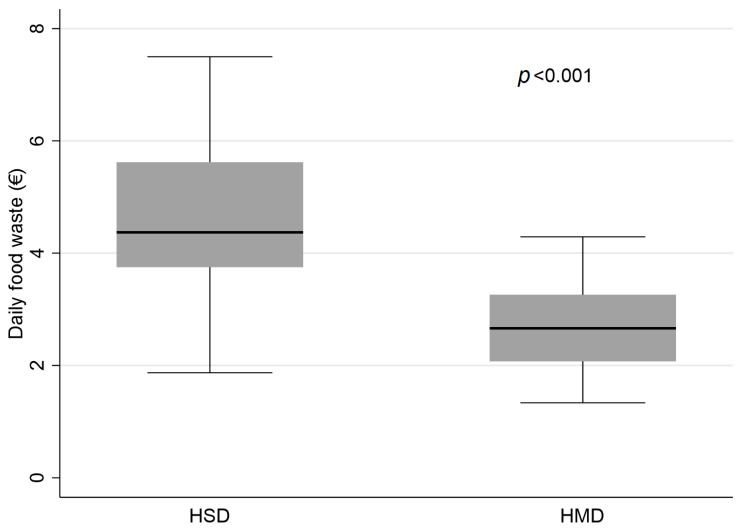
Daily food waste from homogenised diets.

A specific analysis was conducted to evaluate the food waste between the two diets, based on the food not consumed. This evaluation was carried out by calculating the percentage of food consumed (Table 2). From this survey, the daily percentage of food intake was 53.4 ± 12.7% for the HSD diet and 71.6 ± 8.5% for the HMD diet (*p* < 0.001). In support of these results, the daily food waste, derived from the percentage of food eliminated and not eaten, calculated in euros, amounted to EUR 4.66 ± 1.27 for HSD and EUR 2.66 ± 0.81 for HMD (*p* < 0.001) (Figure 4), indicating significantly higher food waste for HSD compared to HMD.

## 4. Discussion 

Given the high prevalence of malnutrition among hospitalized older adult patients, it is crucial to address the underlying drivers of this problem. A significant factor is age-related conditions that prevent regular food intake. As these conditions become more prevalent, there is an ever increasing need to ensure that these patients are provided with adequate nutrition. A common solution for many is a homogenized diet. However, the effectiveness of this diet may be compromised if patients feel it lacks palatability and variety. Historically, hospital settings have offered a rather limited range of homogenized diets which, while nutritionally sound, may not suit the varying taste preferences of the older adult population. Our study, conducted in a tertiary hospital in Northern Italy, aimed to fill this gap. We investigated the comparison between two distinct types of homogenized diets, ensuring that the samples considered were demographically and clinically homogeneous; in this way we were able to eliminate any potential external variable, allowing us to focus exclusively on the properties of the diets themselves. The results underlined the merits of the Homogenized Modified Diet (HMD) when compared to traditional diets like the Homogenized Standard Diet (HSD). Our team’s analysis highlighted that patients on the HMD regimen consumed significantly more calories daily, with a median intake of 852 kcal (IQR 787–926 kcal), contrasting with a median of 631 kcal (IQR 506–797 kcal) for those on the HSD regime. Recognizing appropriate caloric intake is crucial for the overall health and recovery of hospitalized patients. Furthermore, on evaluating rheological attributes such as taste, texture, palatability, and ease of food intake, the HMD consistently surpassed the HSD. Data analysis revealed that the HMD scored a notably higher 3.7 ± 0.6 for taste, indicating a preference between “good” and “very good” on the Likert scale. This result contrasts with the HSD score of 2.5 ± 0.4, which leans more towards “sufficient”. This difference in scores emphasizes the crucial role of dietary palatability in influencing a patient’s food intake, which could subsequently improve the patient’s nutritional status and recovery path. Furthermore, the manner in which the diets were presented emerged as a pivotal factor in the patient experience. The specific packaging of the HMD was designed to optimize the dietary experience, aiming to make meals simpler to consume and more appealing from a sensory perspective. Thus, this feature could significantly impact patient engagement to the proposed dietary regimen. Drawing from our findings, it is apparent that a shift from conventional diets like the HSD to more avant-garde solutions such as the HMD can offer tangible benefits in hospital environments. Notably, our specific analysis highlighted a significant difference in food waste between the two diets. The daily percentage of food intake for the HMD diet was notably higher compared to the HSD diet. This translated to a financial difference with the daily food waste in euros being EUR 4.66 ± 1.27 for HSD and only EUR 2.66 ± 0.81 for HMD, indicating a markedly higher food waste for HSD. This potential advantage of the HMD is not solely from a patient experience or nutritional standpoint, but also manifests in cost-efficiency, further underlined by the significant reduction in food waste.

Lastly, this study contemplated possible biases stemming from clinical variables documented upon hospital admission, including age, BMI, plasma albumin levels, and other comorbidities. It is pivotal to stress that both albumin and the other parameters evaluated recorded consistent values between the two dietary groups at the time of admission. This consistency ensured these elements did not introduce biases. The uniformity observed across these parameters fortified our belief that the conclusions drawn about the advantages of the HMD were not swayed by these considerations.

Our observations align with those presented in a recent systematic review [17], which emphasizes the pressing need for hospital institutions to refine both the nutritional and rheological properties of their food offerings. These considerations target patients requiring modified consistency diets, aiming to mitigate malnutrition risks, prevent weight loss, and enhance the overall meal enjoyment.

### 4.1. Study Limitations 

This observational study has limitations due to the retrospective design adopted. Therefore, we selected the sample based on a specific three-month time frame, without calculating a sample size for the specific study departments. Nevertheless, we believe that the study sample is representative of current hospital dietary practice, reflecting the daily challenges faced by these departments. A second limit, despite the fact that distinct phases of data collection, anonymization of data, and primary data collection by distinct professionals have been applied, could be considered the impossibility of applying blinding to our study design. Another limitation in our study is the exclusive reliance on BMI and serum albumin levels measured at hospital admission to assess nutritional status. Although this approach was relevant to our retrospective study, it may not comprehensively represent nutritional status throughout the entire hospital stay for all patients.

### 4.2. Clinical Implications

In the light of the results of our study, the implementation of more palatable homogenized diets emerges as a possible advantage in reducing hospital malnutrition in terms of both efficacy and efficiency, also contributing to a reduction in food waste.

## 5. Conclusions

The data collected in our study strongly suggest that HMD has significant advantages over HSD in multiple respects. HMD not only had a higher daily calorie intake, but was also rated more favourably by patients in terms of taste, texture, palatability, and convenience. The economic aspect was also important. The adoption of HMD involves a lower daily cost than HSD, a factor of great importance especially in the healthcare sector, where the efficient management of economic resources is essential. Added to this is a significant reduction in food waste, which brings both direct economic benefits and positive environmental implications. Therefore, our study highlighted the potential of the modified homogenized diet as a valid and more efficient alternative to the standard diet in the hospital setting. The implications of these findings are far-reaching. First, the adoption of HMD can improve the quality of life of hospitalized patients, thanks to its higher caloric intake and greater palatability. Furthermore, the use of this diet can lead to considerable economic savings for hospitals, also contributing to a more sustainable management of food resources. Future studies should focus on developing these findings and validating them in different contexts. Another interesting development could be the exploration of other variants of the homogenized diet, with the aim of further optimizing both nutritional intake and patient acceptability. Furthermore, the application of this research could extend beyond the hospital setting. Nursing homes, aged care facilities and other care settings could benefit from adopting the modified homogenized diet. This could represent an optimal solution to improve nutritional intake and the acceptability of meals in many categories of patients, including those suffering from dysphagia or other conditions that make regular food intake difficult. The results of this study aim to favour and stimulate the development of new research in the field of dietetics, to improve and optimize nutrition in all hospital and territorial contexts.

## Figures and Tables

**Table 1:** Demographic and clinical characteristics of the sample.

Characteristics	HSD (*n* = 55)	HMD (*n* = 31)	*p* Value
Age (Years)	82.2 ± 9.8	81.5 ± 11.6	0.991
Sex (M)	28 (50.91%)	12 (41.38%)	0.406
BMI (Kg/m^2^)	22.2 ± 3.5	23.4 ± 5.4	0.406
MUST	0.91 ± 1.27	0.94 ± 1.01	0.621
Albumin (g/L)	29.9 ± 6.2	29.0 ± 4.2	0.571
Disease			
Cancer	8 (14.55%)	1 (3.33%)	0.108
UTI	5 (9.09%)	3 (10.00%)	0.891
Pneumonia	15 (27.27%)	8 (26.67%)	0.952
Sepsis	13 (23.64%)	9 (30.00%)	0.522
Neurodegenerative pathologies	21 (38.18%)	10 (33.33%)	0.657
Comorbidity	34 (61.82%)	20 (66.67%)	0.657
COPD	6 (17.65%)	4 (20.00%)	
Cardiopathy	14 (41.18%)	8 (40.00%)	
Diabetes	10 (29.41%)	7 (35.00%)	
CKD (stages I–IV)	9 (26.47%)	7 (35.00%)	
Comorbidity (**n**)	1 (1–3)	1 (1–3)	0.714
Dysphagia	26 (47.27%)	10 (37.04%)	0.479

Legend: HSD, Standard Homogenised Diet; HMD, Modified Homogenized Diet; UTI, Urinary Tract Infection; COPD, Chronic Diet Obstructive Pulmonary Disease; CKD, Chronic Kidney Disease; MUST, Universal Malnutrition Screening Tool.

**Table 2:** Dietary intake, palatability, and rheological properties of homogenised diets.

Characteristics	HSD (*n* = 55)	HMD (*n* = 31)	*p*
Water intake (mL)	500 (0–2000)	375 (187.5–500)	0.357
Daily calorie intake (kcal)	634 ± 204	862 ± 89	<0.001
Portion (% consumed)	53.4 ± 12.7	71.9 ± 8.5	<0.001
Assessment			
Taste	2.5 ± 0.4	3.7 ± 0.6	<0.001
Textures	2.3 ± 0.4	3.2 ± 0.5	<0.001
Palatability	2.3 ± 0.4	3.2 ± 0.5	<0.001
Practicality	2.0 ± 0.4	3.7 ± 0.4	<0.001

Legend. Data are expressed as mean ± SD or median (range).

## Data Availability

Clinical data are made available upon request by contacting the corresponding author.

## Supplementary Materials

The following supporting information can be downloaded at: https://www.mdpi.com/article/10.3390/nu15224731/s1. STROBE Statement.
