# Supplementary material for: A Comparative Evaluation of the Caloric Intake and Economic Efficiency of Two Types of Homogenized Diets in a Hospital Setting
Source: Nutrients. 2023 Nov 9;15(22):4731. doi: 10.3390/nu15224731 (PMC10675474; doi:10.3390/nu15224731)
Supplement: Supplementary file 1 [file nutrients-15-04731-s001.zip › nutrients-2659742-supplementary.pdf]

STROBE Statement

	Item No	Recommendation	Page n
**Title and abstract**	1	(*a*) Indicate the study's design with a commonly used term in the title or the abstract	Pag. 1
		(*b*) Provide in the abstract an informative and balanced summary of what was done and what was found	Pag.1
**Introduction**			
Background/rationale	2	Explain the scientific background and rationale for the investigation being reported	(main manuscript pag.1-2)
(Objectives	3	State specific objectives, including any prespecified hypotheses	(main manuscript pag.2)
**Methods**			
Study design	4	Present key elements of study design early in the paper	(main manuscript pag.2)
Setting	5	Describe the setting, locations, and relevant dates, including periods of recruitment, exposure, follow-up, and data collection	(main manuscript pag.3-4)
Participants	6	(*a*) Give the eligibility criteria, and the sources and methods of selection of participants	(main manuscript pag.3-4)
Variables	7	Clearly define all outcomes, exposures, predictors, potential confounders, and effect modifiers. Give diagnostic criteria, if applicable	(main manuscript pag.4)
Data sources/ measurement	8*	For each variable of interest, give sources of data and details of methods of assessment (measurement). Describe comparability of assessment methods if there is more than one group	(main manuscript pag.4)
Bias	9	Describe any efforts to address potential sources of bias	(main manuscript pag.4)
Study size	10	Explain how the study size was arrived at	N.A
Quantitative variables	11	Explain how quantitative variables were handled in the analyses. If applicable, describe which groupings were chosen and why	(main manuscript pag.4)
Statistical methods	12	(*a*) Describe all statistical methods, including those used to control for confounding	(main manuscript pag.4)
		(*b*) Describe any methods used to examine subgroups and interactions	(main manuscript pag.4)
		(*c*) Explain how missing data were addressed	N.R
		(*d*) If applicable, describe analytical methods taking account of sampling strategy	(main manuscript pag.4)
		(*e*) Describe any sensitivity analyses	(main manuscript pag.4)
**Results**			
Participants	13*	(a) Report numbers of individuals at each stage of	(main manuscript

		study—eg numbers potentially eligible, examined for eligibility, confirmed eligible, included in the study, completing follow-up, and analysed	pag.4,5)
		(b) Give reasons for non-participation at each stage	(main manuscript pag.4,5)
		(c) Consider use of a flow diagram	N.A
Descriptive data	14*	(a) Give characteristics of study participants (eg demographic, clinical, social) and information on exposures and potential confounders	(main manuscript pag.4,5)
		(b) Indicate number of participants with missing data for each variable of interest	(main manuscript pag.4,5)
Outcome data	15*	Report numbers of outcome events or summary measures	(main manuscript pag.4-9)
Main results	16	(*a*) Give unadjusted estimates and, if applicable, confounder-adjusted estimates and their precision (eg, 95% confidence interval). Make clear which confounders were adjusted for and why they were included	(main manuscript pag.4-9)
		(*b*) Report category boundaries when continuous variables were categorized	(main manuscript pag.4-9)
		(*c*) If relevant, consider translating estimates of relative risk into absolute risk for a meaningful time period	N.A
Other analyses	17	Report other analyses done—eg analyses of subgroups and interactions, and sensitivity analyses	(main manuscript pag.8-9)
**Discussion**			
Key results	18	Summarise key results with reference to study objectives	(main manuscript pag.9)
Limitations	19	Discuss limitations of the study, taking into account sources of potential bias or imprecision. Discuss both direction and magnitude of any potential bias	(main manuscript pag.9-10)
Interpretation	20	Give a cautious overall interpretation of results considering objectives, limitations, multiplicity of analyses, results from similar studies, and other relevant evidence	(main manuscript pag.9-10)
Generalisability	21	Discuss the generalisability (external validity) of the study results	(main manuscript pag.9-10)
**Other information**			
Funding	22	Give the source of funding and the role of the funders for the present study and, if applicable, for the original study on which the present article is based	This research received no external funding.

*Give information separately for exposed and unexposed groups.
